# Solvent-Driven Variation in Phytochemical Profile and Antioxidant Activity of *Melissa officinalis* from Tabasco, Mexico, Revealed by UPLC-QtoF

**DOI:** 10.3390/plants15152275

**Published:** 2026-07-25

**Authors:** Nelly Cristina Aguilar-Sánchez, José Rodolfo Velázquez-Martínez, Minerva Aurora Hernández-Gallegos, Emmanuel Cabañas-García, Yenny Adriana Gómez-Aguirre, Angelica Alejandra Ochoa-Flores, Abraham Gómez-Rivera, María Guadalupe Hernández-Cruz

**Affiliations:** 1 División Académica Multidisciplinaria de Jalpa de Méndez, Universidad Juárez Autónoma de Tabasco, Carretera Estatal Libre Villahermosa Comalcalco Km. 27 S/N, Ranchería Rivera Alta, Jalpa de Méndez 86205, Tabasco, Mexico; drnaguilar1018@gmail.com (N.C.A.-S.); mguadalupe.hernandez@ujat.mx (M.G.H.-C.); 2 División Académica de Ciencias Agropecuarias, Universidad Juárez Autónoma de Tabasco, Carretera Villahermosa-Teapa Km. 25+2 Ranchería La Huasteca 2da Sección, Villahermosa 86298, Tabasco, Mexico; angelica.ochoa@ujat.mx; 3 Unidad Profesional Interdisciplinaria de Ingeniería, Campus Zacatecas-Instituto Politécnico Nacional, Calle Circuito del Gato No. 202, Col. Ciudad Administrativa, Zacatecas 98160, Zacatecas, Mexico; ecabanasg@ipn.mx; 4 SECIHTI-Universidad Autónoma de Aguascalientes—Av. Universidad 940, Ciudad Universitaria, Aguascalientes 20131, Aguascalientes, Mexico; yenny.gomez@edu.uaa.mx; 5 División Académica de Ciencias Básicas, Universidad Juárez Autónoma de Tabasco, Carretera Cunduacán-Jalpa Km. 0.5, Cunduacán 86690, Tabasco, Mexico; abgori@gmail.com

**Keywords:** secondary metabolites, solvent extraction, metabolite profiling, phenolic compounds, antioxidant activity

## Abstract

*Melissa officinalis* is recognized as a rich source of phenolic compounds with antioxidant properties. However, although its phytochemical composition has been extensively investigated, comparative studies integrating phytochemical characterization, antioxidant properties, and LC-MS metabolite profiling across commonly used extraction systems remain limited. This study comparatively evaluated aqueous, hydroacetonic, and hydroethanolic extracts of *M. officinalis* leaves by determining total phenolic (TPC), flavonoid (TFC), and tannin (TTC) contents, antioxidant activity (DPPH, ABTS, and FRAP assays), and metabolite profiling using UPLC-QToF-MS. The hydroethanolic extract exhibited the highest TPC (442.17 mg GAE/g DE), TTC (221.17 mg TAE/g DE), and antioxidant activity, whereas the hydroacetonic extract showed the highest TFC (178.52 mg CE/g DE). UPLC-QToF-MS-based metabolite profiling aided in the tentative annotation of 15 metabolites, including phenolic acids (rosmarinic, gallic, and ellagic acids) and flavonoids (catechin, rutin, quercetin, and apigenin). Among the evaluated conditions, the hydroethanolic and aqueous extracts yielded a higher number of metabolites than the hydroacetonic extract. Our findings demonstrate that solvent selection substantially influences phytochemical recovery, metabolite coverage, and antioxidant properties, providing an integrated framework for selecting extraction systems for phytochemical characterization and antioxidant-oriented applications.

## 1. Introduction

*Melissa officinalis* L. (Lamiaceae), commonly known as toronjil, citronella, or limonera, is a perennial medicinal herb native to the Mediterranean region and currently cultivated in Europe, North America, and Asia [1]. In Mexico, it is also distributed and extensively used in traditional medicine for the treatment of gastrointestinal, liver, and neurological disorders and cancer [2]. Additionally, it has been employed for managing fatigue, dizziness, migraine, intestinal spasms, muscle contractions, neuralgia, diabetes, and bacterial infections [3,4]. These properties are attributed to its chemical composition, which includes metabolites with antioxidant activity, such as glycosides and volatile phenolic components (eugenol), caffeic acid derivatives (rosmarinic acid), flavonoids (cynaroside, cosmosiin, rhamnocitrin, isoquercitrin), phenolic acids (carnosic acid), and triterpenic acids (ursolic and oleanolic acids) [5]. The main studies on the identification and analysis of the chemical composition and bioactive properties of *M. officinalis* have used ethanolic [5] and aqueous [6] extracts. However, for extracts derived from other plant species, differences in composition and activity have been reported to depend on plant variety, the extraction mass-to-volume ratio, and solvent affinity [7]. Additionally, other factors that may affect plant extracts’ bioactivities and phytochemical content include culture conditions, such as light intensity, temperature, nutrient availability, and plant age [8]. Although studies have investigated the phytochemical composition and biological properties of *M. officinalis*, most have focused on individual extraction systems or in specific analytical conditions rather than performing integrated comparative analyses using different extraction systems. Consequently, although these studies have substantially improved our understanding of the phytochemical diversity and biological properties of the species, comparative studies integrating solvent-dependent metabolite recovery, antioxidant properties, and metabolite profiling across different extraction systems remain limited.

Tabasco is a Mexican state located in the southeastern region, covering up to 24,700 km^2^ of Mexican territory. Tabasco state is subdivided into five regions: (1) Chontalpa, (2) Rivers, (3) Sierra, (4) Center, and (5) Pantanos [9]. In the Chontalpa region, in the Nacajuca area, where the Mayan-Chontal indigenous groups live, 232 medicinal plants have been identified, including *M. officinalis* due to its widespread use in traditional medicine for the treatment of conditions related to the nervous and digestive systems, where it is used primarily as an infusion to relieve nervousness, insomnia, stress, and stomach ailments. Its significance extends beyond the therapeutic sphere, as it represents a plant resource linked to cultural identity and the transmission of traditional knowledge across generations [10]. Although *M. officinalis* has been investigated across different geographical regions, cultivation conditions, and extraction strategies [11], variation in the phytochemical composition and biological properties has been reported, emphasizing the importance of studying the extraction processes for phytochemical characterization. In this regard, the plant material collected in Tabasco, Mexico, would provide a regional context for evaluating the performance of different extraction systems within plant species traditionally employed by local communities and cultivated under defined environmental conditions.

Consequently, selecting an appropriate extraction system is important not only for maximizing metabolite recovery and coverage, but also for obtaining extracts that more accurately reflect the potential biological properties of the plant material. Therefore, this study aimed to compare the performance of different extraction systems, including aqueous, hydroacetonic, and hydroethanolic, in terms of total phenolic, total flavonoid, and total tannin contents, as well as the antioxidant properties and metabolite profiles of *M. officinalis* leaves collected in Tabasco, Mexico.

## 2. Results and Discussion

### 2.1. Yield, Total Phenolic Content, Total Flavonoid Content, and Total Tannin Content of Melissa officinalis Extracts of Tabasco

Differences were observed in the appearance of the extracts depending on the extraction solvent. The AE was brown in color, whilst the EE and EAC were dark green. Table 1 shows the extraction yields obtained in this study, which ranged from 23 to 34% and followed the order AE > EAC > EE, with values of 34%, 27%, and 23%, respectively. Statistically significant differences (*p* < 0.05) were observed among the extraction systems employed. Previous reports have demonstrated that the yield and quality of plant extracts are influenced by the extraction method and conditions used to obtain bioactive molecules. Additionally, factors such as sample pretreatment, chemical composition, and plant characteristics also play a significant role in the extraction process [12]. Extraction yield should not be interpreted only as an indicator of extraction efficiency, since different solvent systems can recover different classes of plant metabolites depending on their physicochemical properties. Thus, the differences observed in the extract yield can be attributed to the polarity of each solvent [13,14], which governs the selective solubilization of compounds with varying polarities.

Although the extraction yield was lower for EE, this extraction system showed the highest TPC (442.17 mg GAE/g DE), followed by EAC (391.27 mg GAE/g DE), and AE (257.81 ± 1.57 mg GAE/g DE). These results suggest that the extraction yield and phenolic recovery represent different analytical parameters and should not be interpreted as directly correlated. Despite producing the lowest extraction yield, the EE recovered the highest TPC, suggesting that the intermediate polarity of ethanol–water mixtures provides favorable solvent environments for recovering a broader range of phenolic compounds. Additionally, this extraction mixture may reduce the coextraction of highly polar non-phenolic compounds in the extract, compared with aqueous extraction. In previous studies, the maceration process has been shown to extract higher amounts of phenolics, which are associated with the antioxidant properties of plant extracts [12]. Previously, Stini et al. [15] analyzed the TPC in *M. officinalis* byproducts and reported a value of 111 mg GAE/g of dried herb, which is lower than the values obtained in the present study for all extracts. Such variation among studies is more associated with plant genotype, cultivation conditions, extraction methodology, and analytical procedures, all of which are recognized to influence phenolic recovery [16]. For *M. officinalis* cultivars such as Lorelei and Gold Leaf, Szabó et al. [17] reported TPC values of 359 mg GAE/g DE and 427 mg GAE/g DE, respectively, which are similar to those found in this study for EE and EAC. Furthermore, the TPC values found in AE (257.81 mg GAE/g DE) were similar to those reported by Wojdylo et al. [18] and Dastmalchi et al. [19].

Flavonoids are metabolites recognized for their biological activities [20]. In this study, the EAC showed the highest TFC (178.52 mg CE/g DE), followed by EE and AE (145.55 and 82.34 mg CE/g DE, respectively). This suggests that the hydroacetonic extraction system provides conditions for recovering specific flavonoid subclasses, further demonstrating that solvent system selection influences not only the amount but also the composition of the extracted phenolic fraction. These values were higher than those reported for other Lamiaceae species such as *Origanum majorana* L. originating from Serbia, Greece, Egypt, and Libya [21], with values ranging from 11.03 to 15.64 mg quercetin equivalent of dry extract (mg QE/g DE) in aqueous extracts and 14.87 to 20.05 mg QE/g DE in ethanolic extracts.

Another class of polyphenolic metabolites with reported pharmacological properties is tannins [22]. In our research, the EE showed the highest tannin content (221.17 mg TAE/g DE), followed by AE (153.28 mg TAE/g DE) and EAC (83.25 mg TAE/g DE; see Table 1), consistent with the TPC; the greater tannin recovery obtained with the EE system further supports the influence of solvent composition on the selective extraction of different phenolic classes. Total tannin content has been reported for plant extracts prepared with other Lamiaceae species, with lower values. Kpètèhoto et al. [23] reported 8.6 mg TAE/100 g DE of total tannin content in ethanolic extracts of *Ocimum gratissimum* L. Similarly, Moshari-Nasirkandi et al. [24] reported the TTC in ethanolic extracts prepared with 20 different plant species of the Lamiaceae family, ranging from 0.33 to 6.49 mg TAE/100 g DE. Collectively, these findings suggest that the extraction system influences the recovery of different classes of phenolic compounds rather than uniformly increasing the extraction of all metabolites. Hence, the selection of an appropriate solvent system should be guided by the intended phytochemical or analytical objective to maximize the recovery of specific metabolites or to obtain extracts with broader phytochemical coverage for subsequent characterization. Thus, the combined measurement of TPC, TTC, and TFC across different plant sources enables the identification of candidates for extracting specific metabolite classes.

### 2.2. Antioxidant Activity

For antioxidant activity analysis, three mechanisms have been suggested by which phenolic compounds can scavenge free radicals: (1) hydrogen atom transfer, (2) single electron transfer, and (3) sequential electron transfer [25]. The DPPH assay combines the hydrogen-atom-transfer mechanism and single-electron-transfer mechanism [26]. In the DPPH assay, the dose of antioxidants in the extract or reference standard required to inhibit the initial activity of the DPPH radical by 50% is known as the IC_50_ value [27]; hence, the lower the IC_50_ value, the higher the antioxidant activity of the extracts. On the other hand, ABTS^•+^ and FRAP are single-electron-transfer methods that measure an antioxidant’s ability to transfer an electron to reduce metal ions, carbonyl groups, and free radicals. Together with DPPH, these methods enable a more comprehensive assessment of the antioxidant activity of plant extracts [28,29,30], allowing the antioxidant response to be interpreted in relation to the phytochemical composition recovered by different extraction systems.

The EE exhibited the strongest DPPH scavenging activity, with the lowest IC_50_ value (78.50 μg/mL). Although this extract showed the lowest yield, it also exhibited the highest TPC and TTC. The superiority of the EE in the DPPH, ABTS, and FRAP assays suggests that the enhanced antioxidant performance is associated with the greater recovery of phenolic constituents rather than with extraction yield itself. Among the extracts, AE showed the weakest activity (132.24 μg/mL), while EAC demonstrated moderate activity (IC_50_ = 107.37 μg/mL; see Table 2). For the ABTS method, the antioxidant activity showed a similar trend: EE > EAC > AE, with values of 154.60 µg/mL, 205.43 µg/mL, and 251.57 µg/mL, respectively. On the other hand, the FRAP assay EE showed the highest antioxidant activity (6.02 ± 0.39 mM TE/g DE), followed by EAC and AE (5.45 ± 0.01 and 3.46 ± 0.05 mM TE/g DE, respectively).

Different studies have analyzed the antioxidant activity of *M. officinalis* extracts. For instance, Kamdem et al. [31] evaluated the antioxidant activity of ethanolic extracts, reporting an IC_50_ of 48.76 ± 1.94 μg/mL, which is similar to the EE values observed in our work. Similarly, Koksal et al. [32] evaluated the antioxidant activity of aqueous and ethanolic extracts prepared from *M. officinalis*, with the aqueous extract showing the highest activity (IC_50_ of 31.4 µg/mL) and the ethanolic extract showing lower activity (IC_50_ of 202.7 µg/mL). This is lower than the activities observed in our study. On the other hand, Souihi et al. [33] evaluated different populations of *M. officinalis*, reporting the highest antioxidant activity in the ethanolic extract, with IC_50_ values ranging from 123.29 ± 0.22 to 541.81 ± 0.33 mg/mL, and from 340.28 ± 0.27 to 645.57 ± 0.32 mg/mL, IC_50_ in the aqueous extract. The variability reported among studies likely reflects the combined influence of plant genotype, cultivation and postharvest conditions, extraction methodology, and analytical procedures, emphasizing that antioxidant performance is determined by multiple experimental factors rather than a single variable.

The extraction of phenolic compounds from *M. officinalis* has been predominantly carried out using aqueous and hydroethanolic solvents, which generally exhibit higher extraction efficiency and antioxidant activity. In contrast, hydroacetonic systems have been scarcely explored, and the limited evidence available suggests a comparatively lower total phenolic content and reduced antioxidant capacity relative to ethanolic extracts [19]. Notably, in the present study, the hydroacetonic extract (EAC) exhibited moderate total phenolic content (TPC) and antioxidant activity, surpassing those of the aqueous extract (AE). This behavior contrasts with previously reported trends, where aqueous systems are often associated with higher antioxidant performance.

### 2.3. Correlation Analysis Between Antioxidant Activity and Total Phenolic Content (TPC)

Phenolic metabolites have a close relationship with the antioxidant activity of plant extracts, and this is generally confirmed by the correlation between the total phenolic content and the antioxidant activity of the extracts, obtaining values that, when closer to 1, indicate a stronger association between phenolic content and antioxidant activity [24,30,34]. Negative values in the correlation coefficient indicate that the lower the IC_50_ value, the greater the antioxidant activity. Therefore, correlation analysis provides additional tools for interpretation by evaluating whether variations in antioxidant performance are consistent with differences in phenolic recovery among the evaluated extraction systems.

The Pearson correlation coefficient between the antioxidant activity assays and the TPC in all extracts is shown in Table 3. A strong negative correlation was observed between TPC and the DPPH antioxidant assay (r = −0.864) and between TPC and ABTS^•+^ analysis (−0.911), indicating that a lower IC_50_ value corresponds to higher antioxidant activity. On the other hand, regarding the correlation between TPC and the FRAP assay, a strong positive relationship was observed (r = 0.978), indicating that higher TPC values are associated with greater reducing power. Collectively, these correlations demonstrate a strong association between phenolic recovery and antioxidant performance across the evaluated extraction systems. Although TPC analysis does not capture the contribution of individual metabolites or potential synergistic interactions, the observed relationships support the use of TPC as a reliable indicator of the antioxidant potential of the evaluated extraction conditions evaluated in this study.

### 2.4. LC-MS Analysis

The effect of the extraction system on the phytochemical profile of *M. officinalis* extracts was analyzed using UPLC-QToF, which enabled the tentative annotation of secondary metabolites based on accurate mass measurements and comparison with spectrometric evidence. The chromatographic profile is presented in Figures S1–S3. The results suggest that solvent selection influenced metabolite coverage, with each extraction system yielding a distinct phytochemical profile rather than uniformly recovering the same metabolites. Depending on the extraction system employed, different metabolites were observed. The highest metabolite coverage was observed in EE and AE, with 10 metabolites of different classes, mainly phenolic acids and flavonoids (see Table 4). The tentatively annotated metabolites in both EE and AE included gallic acid, catechin, quercetin, caffeic acid, rosmarinic acid, and myricetin. In addition, glaucolactone was detected exclusively in the EE, for which, to the best of our knowledge, no previous reports describing the presence of this compound have been reported in *M. officinalis*. On the other hand, in the EAC, two flavonoids (myricetin and apigenin) along with rosmarinic acid, pentaerythritol tetrapropionate, and hecogenin acetate have been reported, with the latter two compounds not having been previously described in *M. officinalis*. These findings indicate that solvent selection influences not only the number of metabolites but also the phytochemical coverage obtained from the same plant material, highlighting the importance of extraction conditions for comprehensive phytochemical characterization. Our findings suggest that AE and EE are the extraction solvents that yield the greatest metabolite coverage under the analytical conditions evaluated. However, because metabolite assignments were based on accurate-mass database annotation, confirmation using authentic standards and complementary structural approaches will be necessary to unequivocally establish the identity of these compounds, as well as quantitative approaches. This behavior is likely associated with differences in solvent polarity, which affect the solubility and extraction efficiency of metabolites with different chemical structures and polarities. Consequently, aqueous and hydroethanolic systems were associated with broader phenolic constituent recovery, whereas the hydroacetonic system yielded a more selective phytochemical profile.

Several studies have reported that the main bioactive constituents in species of the Lamiaceae family include terpenoids, phenolic acids, and flavonoids [24,35]. Among phenolics, rosmarinic acid is reported to be one of the most abundant metabolites in Lamiaceae species [24]. This metabolite has also been reported to have therapeutic potential for reducing diseases caused by stress or bacterial infections. In this regard, Abdellatif et al. [4] proposed that rosmarinic acid constitutes 55.39% of *M. officinalis* aerial parts. In our research, rosmarinic acid was tentatively annotated in all extraction systems. Its occurrence across all extraction systems, despite differences in overall metabolite profiles, suggests that this metabolite is a characteristic phenolic constituent of *M. officinalis*; meanwhile, the recovery of other metabolites appears to be more dependent on solvent composition.

## 3. Materials and Methods

### 3.1. Plant Material

Leaves of *Melissa officinalis* were collected in the municipality of Jalapa, Tabasco, Mexico (17°49′18.2″ N, 92°47′44.6″ W), during the harvest in October 2017. The collected leaves were washed with tap water, and the excess moisture was removed using a manual centrifuge. Finally, the leaves were dried for five days under dark conditions at room temperature (30 ± 2 °C). The dried leaves were ground using a mechanical grinder, and the resulting powder was sieved through a No. 60 mesh. The powder was then stored at 4 °C in amber-colored flasks.

### 3.2. Sample Preparation

The extracts were prepared by mixing 5 g of dry powder with 100 mL of each extraction solvent. The aqueous extract (AE) was prepared by heating the mixture at 90 °C for 10 min in a thermobath with shaking. The hydroacetonic extract (EAC) was prepared by mixing deionized water and acetone (70:30 *v*/*v*), and the hydroethanolic extract (EE) was prepared by mixing ethanol and deionized water (70:30 *v*/*v*). The hydroethanolic and hydroacetonic extracts were prepared by maceration for 24 h at 150 rpm in an orbital shaker (DLAB Scientific Co., Ltd., Beijing, China). The aqueous extract was filtered through Whatman No. 1 paper and then through a nitrocellulose membrane (0.45 μm) using Millipore equipment (Merck Millipore, Burlington, MA, USA). and was dehydrated by freeze-drying (lyophilization). The hydroacetonic and hydroethanolic extracts were filtered through Whatman No. 1 paper and were centrifuged at 4000× *g*; the supernatants were recovered. Both supernatants were concentrated by rotatory evaporation BUCHI Rotavapor™ R-210 (BUCHI Labortechnik AG, Flawil, Switzerland) at 50 °C, and excess moisture was removed by drying the samples in an oven at 50 °C. The resulting extract (1.2% humidity) was stored at 4 °C in the dark until analysis. Prior to the spectrophotometric determinations, each dried extract was dissolved in the same solvent used for its extraction at a concentration of 10 mg/mL. Subsequently, appropriate dilutions were prepared as required for each assay to obtain absorbance values within the linear range of the corresponding calibration curves.

### 3.3. Total Phenolic Content (TPC)

TPC was quantified using the Folin–Ciocalteu spectrophotometric method proposed by Singleton et al. [36], with slight modifications for the present study. For each extract, a mixture was prepared with 125 µL of the extract and 625 µL of Folin–Ciocalteu reagent (HYCEL, Jalisco, Mexico) diluted 1:10. Then, 500 µL of 7.5% Na_2_CO_3_ (J.T. Baker, Ecatepec, Estado de México, Mexico) was added, and the mixture was allowed to react for 45 min in dark conditions. The absorbance of the reaction was measured at 760 nm. For data analysis, a standard reference curve of gallic acid (Sigma-Aldrich, St. Louis, MO, USA) was prepared, ranging from 10 to 100 mg/mL. The results were reported as milligrams of gallic acid equivalent per gram of dry extract (mg GAE/g DE). All determinations were performed in triplicate.

### 3.4. Total Flavonoid Content (TFC)

TFC was determined using the methodology described by Todaro et al. [37], with slight modifications for the present study. For each extract, a mixture was prepared with 500 µL of extract, 2 mL of distilled water, and 150 µL of NaNO_2_ (5%) (J. T. Baker, MX). The mixture was stirred and allowed to react for 6 min. Subsequently, 150 µL of AlCl_3_ (10%) (J. T. Baker, MX) was added, and the mixture was allowed to stand for another 6 min. Then, the reaction was treated with 800 µL of NaOH (10%) (J. T. Baker, MX), and the mixture was adjusted to a final volume of 5 mL with distilled water. For the analysis of the obtained data, a catechin (Sigma-Aldrich, St. Louis, MO, USA) reference curve ranging from 10 to 100 CE/µL was prepared. The absorbance of the reaction was measured at 510 nm. All measurements were performed in triplicate, and results were reported as milligrams of catechin equivalent per gram of dry extract (mg CE/g DE).

### 3.5. Total Tannin Content (TTC)

The TTC determination was performed as proposed by Makkar [38], Ramos et al. [39], and Chaijan et al. [40]. A 2 mL volume of each extract with known TPC concentrations was homogenized with 100 mg of polyvinylpolypyrrolidone (PVPP, Sigma-Aldrich, St. Louis, MO, USA) and allowed to react for 15 min at 4 °C. After the reaction time, the mixture was centrifuged at 3000× *g* for 10 min. The TTC was calculated by comparing the samples’ TPC with the TPC after reaction with PVPP. All measurements were performed in triplicate, and the total tannin content was expressed as milligrams of tannic acid equivalents per gram of dry extract (mg TAE/g dry extract).

### 3.6. Antioxidant Activity

#### 3.6.1. DPPH Free Radical Scavenging Activity

For DPPH (2,2-diphenyl-1-picrylhydrazyl, acid; Sigma-Aldrich, St. Louis, MO, USA) free radical scavenging activity, a 0.125 mM DPPH solution in 80% methanol was prepared. Then, 200 μL of each extract at a previously adjusted concentration was mixed with 2 mL of the DPPH• solution. The mixture was allowed to react in the dark at room temperature (25 ± 2 °C) for 60 min. The antioxidant capacity was evaluated by measuring the absorbance of the DPPH• solution and the mixture at 520 nm. The DPPH inhibition percentage was calculated using the formula: % inhibition: (Abs_control_ − Abs_sample_)/(Abs_control_) × 100. Additionally, the IC_50_ value (the extract concentration required to inhibit 50% of the radicals) was calculated using linear regression analysis, as proposed by Sánchez-Zarate et al. [41].

#### 3.6.2. ABTS^•+^ Radical Scavenging Assay

The ABTS^•+^ radical-scavenging assay was performed using the improved ABTS radical cation decolorization assay, as reported by Re et al. [42], with slight modifications for the present study. The ABTS^•+^ radical solution was prepared by reacting 2.45 mM potassium persulfate (Sigma-Aldrich, St. Louis, MO, USA) with 7 mM ABTS^•+^ (1:1) and incubating the mixture for 16 h at room temperature (25 ± 2 °C) in the dark. The absorbance of the ABTS^•+^ solution was adjusted with methanol to obtain an absorbance of 0.700 at 734 nm. The ABTS^•+^ radical scavenging activity of extracts was analyzed by mixing 10 μL of each extract at a previously adjusted concentration with 990 μL of the adjusted solution. The mixture was incubated for 6 min, and then the absorbance was read at 734 nm. The results were reported as IC_50_ (μg/mL), calculated using the same procedure as in the DPPH assay.

#### 3.6.3. Ferric Reducing Antioxidant Power (FRAP) Assay

The FRAP assay was performed as reported by Sánchez-Zarate et al. [41]. For instance, the FRAP solution was prepared by mixing 300 mM acetate buffer, pH 3.6, 10 mM TPTZ (1,3,5-triazine, 2,4,6-tri-2-pyridinyl-; Sigma-Aldrich, St. Louis, MO, USA) in 40 mM HCl (J. T. Baker, MX), and 20 mM FeCl (J. T. Baker, MX) in a ratio of 10:1:1 at room temperature. Subsequently, 1200 µL of the FRAP solution was taken and mixed with 40 μL of each extract and 120 μL of distilled water. The reaction mixture was incubated for 30 min at room temperature (25 ± 2 °C), and then the absorbance was monitored at 593 nm. Trolox (Sigma-Aldrich, St. Louis, MO, USA) was used as a reference standard, and a calibration curve was prepared to estimate the antioxidant activity. Results were reported as mM Trolox equivalents per gram of dry extract (mM TE/g DE).

### 3.7. LC-MS Analysis

The metabolite profiling of *M. officinalis* leaf extracts was carried out using UPLC-QToF equipment, as previously reported by Sánchez-Zarate et al. [41]. One gram of each dried sample was resuspended in 1.5 mL of methanol (J.T. Baker, MX) and then sonicated for 10 min. The dissolved material was subjected to solid-phase extraction with a C18 column using methanol (SUPELCO™) as the mobile phase. Once the eluate was obtained, the remaining solvent was removed at 40 °C under reduced pressure using a rotary evaporator (BUCHI Rotavapor™ R-210). Next, 100 mg of each extract was resuspended in methanol acidified with 0.1% formic acid, sonicated for 5 min, and filtered using a 0.2 μm nylon membrane.

For chromatographic separation and mass spectrometric analysis, a Waters^®^ Acquity UPLC^®^ Class I system (C18, 2.1 × 100 mm and 1.8 μm column) hyphenated to a Xevo^®^ G2 XS Qtof mass spectrometer was employed. The chromatographic separation was performed in a gradient elution program composed of a mixture of water:acetic acid (99.9:0.1 *v*/*v*, solvent A) and acetonitrile (100%, solvent B) as follows: 97% A and 3% B (0–30 min), 3% A and 97% B (30–32 min), 97% A and 3% B (32–35 min). The flow rate was set at 0.2 μL/min, and the injection volume was 5 μL. The mass spectrometer system was equipped with an electrospray ionization source (Waters Corp., Milford, MA, USA) and operated in negative ionization mode (ESI-) and sensitivity mode, with the mass range set to *m*/*z*: 100–1200. As a reference standard, leucine enkephalin (*m*/*z*: 554.2615) was used. For data acquisition and processing, MassLynx software V. 4.1 and Progenesis QI (Waters Corporation, Milford, MA, USA) were used. Candidate molecular formulas were generated using Progenesis QI, and the corresponding theoretical monoisotopic masses were calculated with the Molecular Weight Calculator implemented in MassLynx V4.1. Tentative metabolite annotation was performed by comparing accurate mass measurements (mass tolerance ≤ 20 ppm) and the proposed molecular formulas with information available in specialized chemical and phytochemical databases, including NIST, NIST Chemistry WebBook, MassBank, Natural Product Updates, Natural Remedies, Plant Metabolic Network, The National Compound Collection, Phenol-Explorer, and the SDBS Spectral Database for Organic Compounds, together with previously published literature. Candidate structures were proposed based on the highest agreement between accurate mass measurements, molecular formula predictions, database searches, and previously reported phytochemical information. Accordingly, all metabolites reported in this study are presented as tentative annotations based on accurate-mass database-assisted analysis.

### 3.8. Statistical Analysis

All tests were performed in triplicate. The results were evaluated using a one-way analysis of variance, and the means were compared using Tukey’s multiple range test (*p* < 0.05). The correlation between antioxidant activity and total phenolic content of the extracts was assessed using Pearson’s correlation coefficient (*p* < 0.05). Statistical analyses were conducted using IBM SPSS Statistics v22 software.

## 4. Conclusions

*Melissa officinalis* is a phenolic-rich species with well-documented biological activities. However, *M. officinalis* cultivated in Tabasco, Mexico, exhibited marked differences in phytochemical composition and antioxidant performance depending on the extraction system employed. The hydroethanolic extract (EE) exhibited the highest contents of total phenols, flavonoids, and tannins, as well as the greatest antioxidant activity in the DPPH, ABTS, and FRAP assays. Furthermore, a strong correlation exists between total phenolic content and antioxidant capacity in the extracts. Pearson correlation coefficients indicate that higher phenolic content is associated with lower IC_50_ values in the DPPH and ABTS assays (indicating greater antioxidant activity) and greater reducing capacity in the FRAP assay. UPLC-QToF analysis enabled the tentative annotation of metabolites, primarily phenolic acids and flavonoids, in the extracts. The EE and the aqueous extract (AE) provided the broadest metabolite coverage, including phenolic acids (rosmarinic, gallic, ellagic, caffeic) and flavonoids (catechin, quercetin, myricetin, rutin). In addition, compounds such as glaucolactone, pentaerythritol tetrapropionate, and hecogenin acetate were tentatively annotated, for which no previous reports in *M. officinalis* were identified in the literature. Our findings suggest that solvent selection strongly influences metabolite recovery, metabolite coverage, and hence, antioxidant performance. The hydroethanolic extraction system provided the most comprehensive recovery of antioxidant-related phenolic constituents under the evaluated analytical conditions. Our results also provide a methodological basis for selecting extraction systems according to specific phytochemical and analytical objectives and contribute to the phytochemical characterization of *M. officinalis* cultivated in Tabasco, Mexico.

## Figures and Tables

**Table 1:** Effect of the extraction systems on the extract yield, total phenolic content (TPC), total flavonoid content (TFC), and total tannin content (TTC) of *Melissa officinalis* aqueous extract (AE), hydroacetonic extract (EAC), and hydroethanolic extract (EE). The values represent the average (*n* = 3) ± the standard deviation. The same letters within columns indicate that there are no statistical differences among the extraction systems (*p* < 0.05).

Extract	Yield of the Extract %	TPC (mg GAE/g DE)	TFC (mg CE/g DE)	TTC (mg TAE/g DE)
AE	34 ± 1.05 ^a^	257.81 ± 1.57 ^c^	82.34 ± 15.35 ^c^	153.28 ± 4.59 ^b^
EAC	27.2 ± 0.89 ^b^	391.27 ± 38.53 ^b^	178.52 ± 25.60 ^a^	83.25 ± 4.19 ^c^
EE	23 ± 0.75 ^c^	442.17 ± 12.26 ^a^	145.55 ± 27.50 ^b^	221.17 ± 31.14 ^a^

**Table 2:** Effect of the extraction systems on the antioxidant activity (DPPH, ABTS^•+^, and FRAP) of *Melissa officinalis* L. extracts. The values represent the average (n = 3) ± the standard deviation. The same letters within columns indicate that there are no statistical differences among the extraction solvents (*p* < 0.05).

Extract	DPPH (µg/mL, IC_50_)	ABTS^•+^ (µg/mL, IC_50_)	FRAP (mM TE/g DE)
AE	132.24 ± 14.78 ^a^	251.57 ± 9.94 ^a^	3.46 ± 0.05 ^a^
EAC	107.37 ± 6.96 ^b^	205.43 ± 8.21 ^b^	5.45 ± 0.01 ^b^
EE	78.50 ± 1.54 ^c^	154.60 ± 8.17 ^c^	6.02 ± 0.39 ^b^

**Table 3:** Correlation studies of antioxidant activity measured by different methods and TPC of *Melissa officinalis* extracts.

Assay	Equation	Pearson Correlation (r^2^)	*p* Value
DPPH (IC_50_)	y = 198.19 − 0.249x	−0.864	*p* < 0.01 (*p* = 0.003)
ABTS (IC_50_)	y = 371.84 − 0.454x	−0.911	*p* < 0.01 (*p* ≤ 0.01)
FRAP (mM TE/g DE)	y = 0.21 + 0.14x	0.978	*p* < 0.01 (*p* ≤ 0.01)

**Table 4:** Tentatively identified metabolites detected in *Melissa officinalis* extracts by UHPLC-QTOF-MS.

Extract	Tentatively Identified Metabolite	Compound Class	Retention Time (min)	Elemental Composition	Measured Mass [M-H]	Theoretical Mass [M-H]	Mass Error (ppm)
AE	Ellagic acid	Phenolic acid	6.9	C_14_H_6_O_8_	300.9984	300.9990	1.99
	Gallic acid	Phenolic acid	3.0	C_7_H_6_O_5_	169.0137	169.0142	2.96
	Catechin	Flavonoid (flavanol)	1.2	C_15_H_14_O_6_	289.0712	289.0718	2.08
	Rosmarinic acid	Phenolic acid	10.4	C_18_H_16_O_8_	359.0767	359.0772	1.39
	Quercetin	Flavonoid (flavonol)	13.3	C_15_H_10_O_7_	301.0348	301.0354	1.99
	Quercitrin	Flavonoid (flavonol)	4.0	C_21_H_20_O_11_	447.0927	447.0933	1.34
	Caffeic acid	Phenolic acid	1.9	C_9_H_8_O_4_	179.0344	179.0350	3.35
	Myricetin	Flavonoid (flavonol)	6.2	C_15_H_10_O_8_	317.0297	317.0303	1.89
	Rutin	Glycosylated Flavonoid	5.1	C_27_H_30_O_16_	609.1570	609.1461	17.89
EAC	Myricetin	Flavonoid (flavonol)	6.2	C_15_H_10_O_8_	317.0297	317.0303	1.89
	Apigenin	Flavonoid (flavone)	6.8	C_15_H_10_O_5_	269.0512	269.0455	21.19
	Rosmarinic acid	Phenolic acid	10.4	C_18_H_16_O_8_	359.0767	359.0772	1.39
	Pentaerythritol tetrapropionate	Ester	14.0	C_17_H_28_O_8_	359.1706	359.1706	0.00
	Hecogenin acetate	Sapogenin	22.5	C_29_H_44_O_5_	471.3110	471.3110	0.00
EE	Gallic acid	Phenolic acid	3.0	C_7_H_6_O_5_	169.0137	169.0142	2.96
	Catechin	Flavonoid (flavanol)	1.2	C_15_H_14_O_6_	289.0712	289.0718	2.08
	Luteolin	Flavonoid (flavone)	13.4	C_15_H_10_O_6_	285.0399	285.0405	2.10
	Quercetin	Flavonoid (flavonol)	13.3	C_15_H_10_O_7_	301.0348	301.0354	1.99
	Caffeic acid	Phenolic acid	1.2	C_9_H_8_O_4_	179.0344	179.0350	3.35
	Myricetin	Flavonoid (flavonol)	6.2	C_15_H_10_O_8_	317.0297	317.0303	1.87
	Apigenin	Flavonoid (flavone)	6.8	C_15_H_10_O_5_	269.0512	269.0455	21.19
	Rosmarinic acid	Phenolic acid	10.4	C_18_H_16_O_8_	359.0767	359.0772	1.39
	Hecogenin acetate	Sapogenin	22.5	C_29_H_44_O_5_	471.3110	471.3110	0.00
	Glaucolactone	Terpene	27.4	C_29_H_44_O_4_	455.3161	455.3167	1.27

## Data Availability

The original contributions presented in this study are included in the article/Supplementary Materials. Further inquiries can be directed to the corresponding authors.

## Supplementary Materials

The following supporting information can be downloaded at: https://www.mdpi.com/article/10.3390/plants15152275/s1, Figure S1: UPLC-QToF chromatogram of the aqueous extract (TA) of *Melissa officinalis* L.; Figure S2: UPLC-QToF chromatogram of the hydroethanolic extract (TE) of *Melissa officinalis* L.; Figure S3: UPLC-QToF chromatogram of the hydroacetonic extract (TC) of *Melissa officinalis* L.
