# Supplementary material for: Solvent-Driven Variation in Phytochemical Profile and Antioxidant Activity of Melissa officinalis from Tabasco, Mexico, Revealed by UPLC-QtoF
Source: Plants (Basel). 2026 Jul 25;15(15):2275. doi: 10.3390/plants15152275 (PMC13468195; doi:10.3390/plants15152275)
Supplement: Supplementary file 1 [file plants-15-02275-s001.zip › plants-4398480-supplementary.pdf]

Supplementary Materials

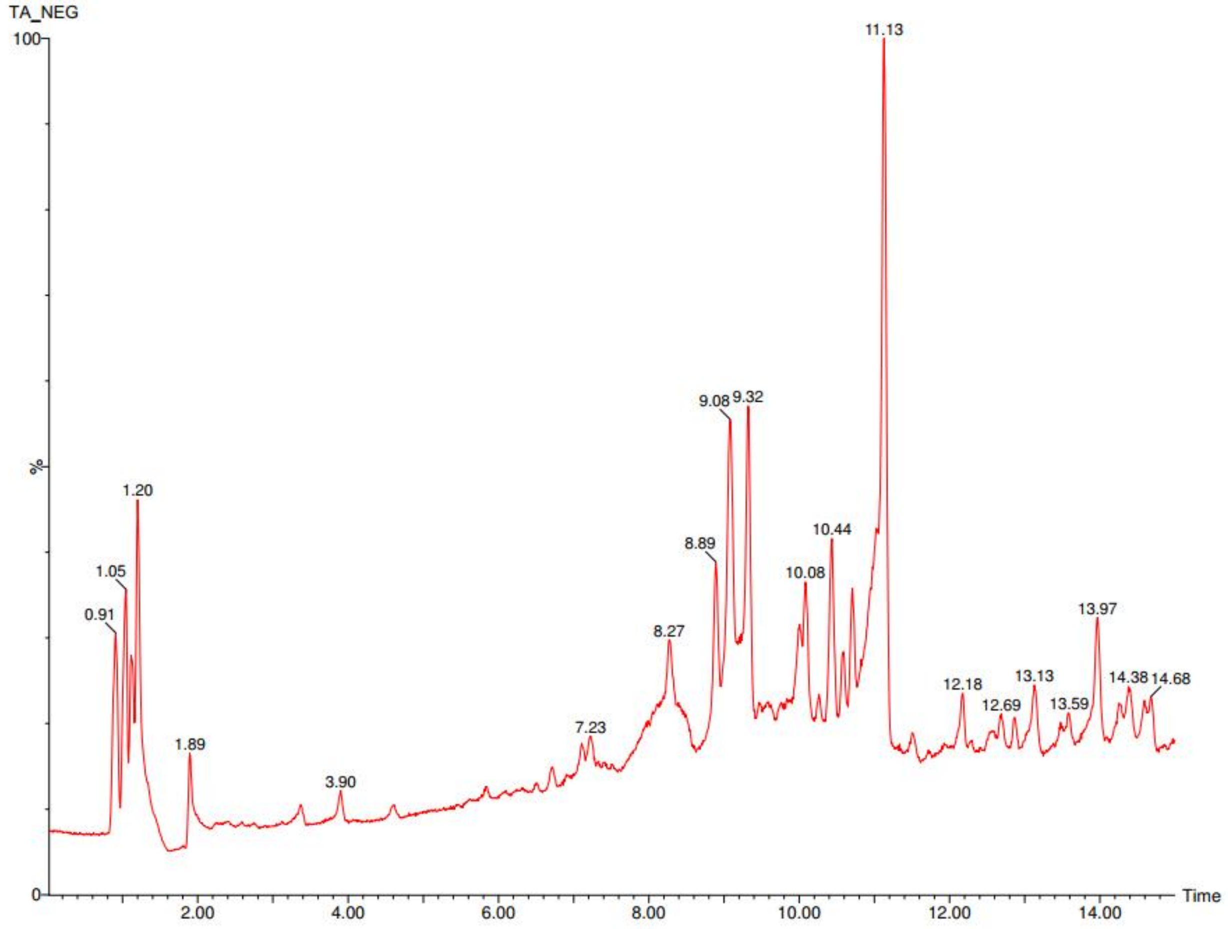

Supplementary Figure S1. UPLC-QToF chromatogram of the aqueous extract (TA) of *Melissa officinalis L.*

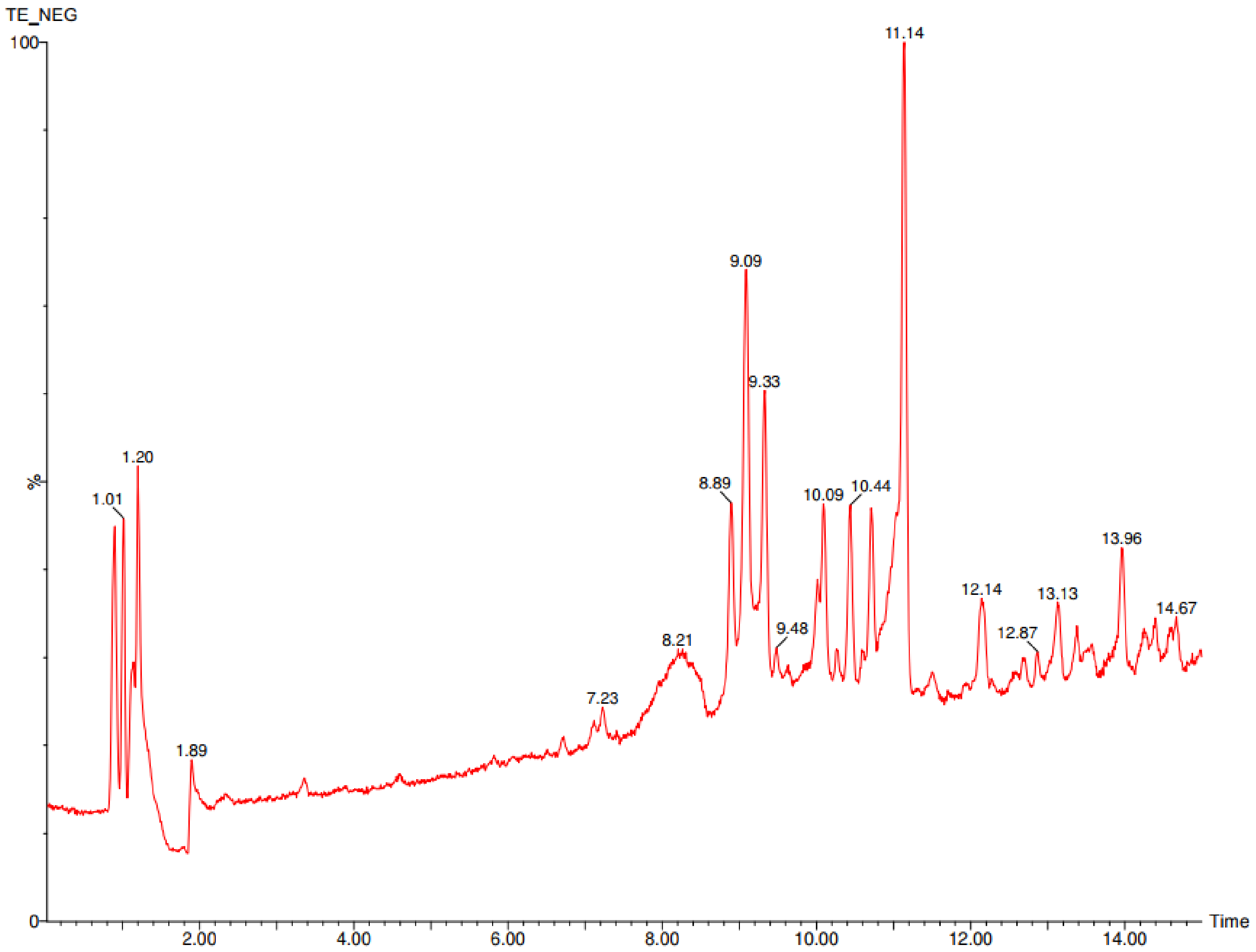

Supplementary Figure S2. UPLC-QToF chromatogram of the hydroethanolic extract (TE) of *Melissa officinalis L.*

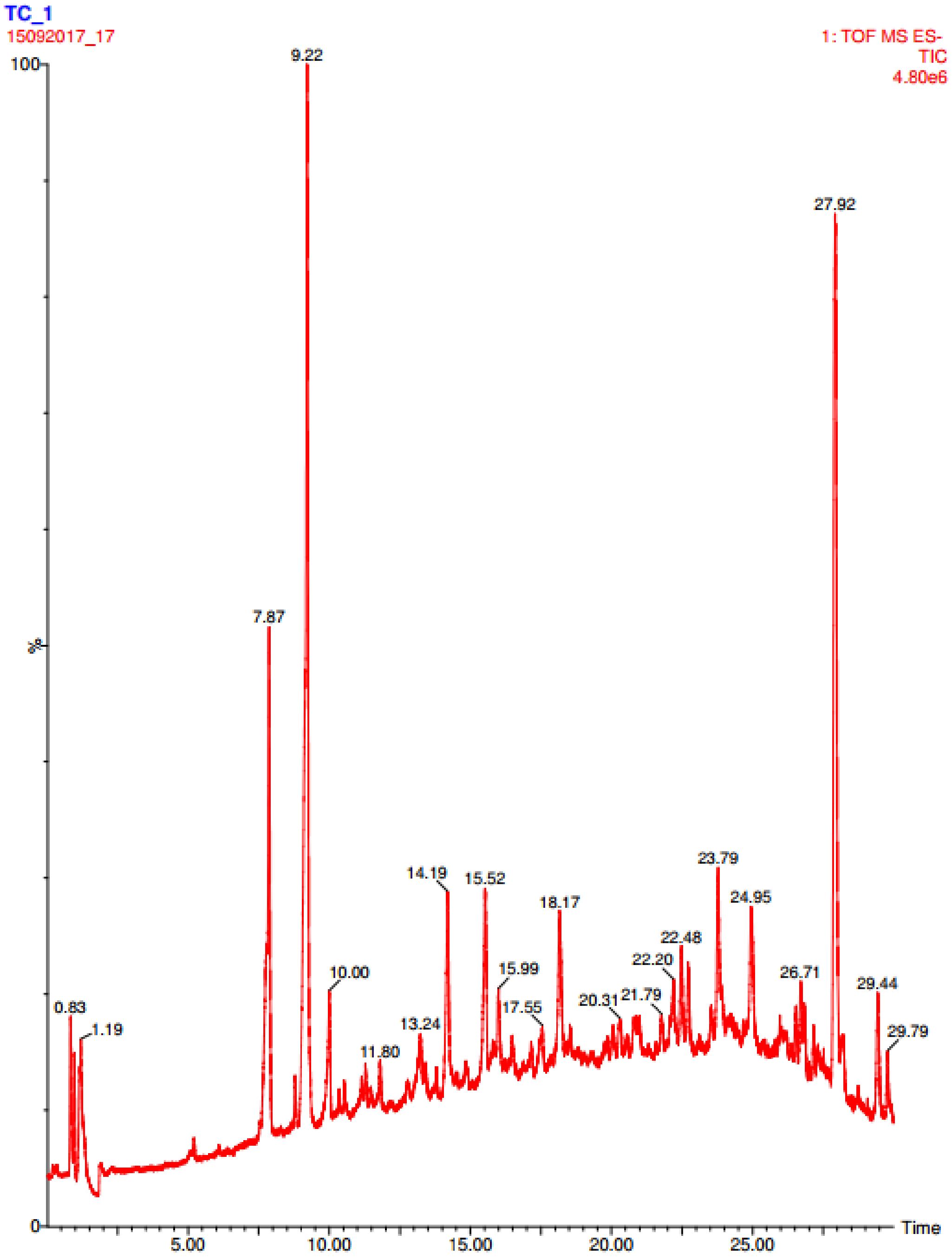

Supplementary Figure S3. UPLC-QToF chromatogram of the hydroacetonic extract (TC) of *Melissa officinalis L.*

Methodological Note

Chromatographic profiles were obtained by UPLC-QToF analysis as described in the Materials and Methods section of the manuscript. The chromatograms are provided as supplementary information to support the qualitative identification of secondary metabolites and to facilitate comparison of the phytochemical profiles of extracts obtained using solvents of different polarities.

Citation of Supplementary Materials

Supplementary Figure S1: UPLC-QToF chromatogram of the aqueous extract (TA) of Melissa officinalis L.; Supplementary Figure S2: UPLC-QToF chromatogram of the hydroethanolic extract (TE) of Melissa officinalis L.; Supplementary Figure S3: UPLC-QToF chromatogram of the hydroacetonic extract (TC) of Melissa officinalis L.
